# Molecular-Signaling Pathways of Ginsenosides Rb in Myocardial Ischemia-Reperfusion Injury: A Mini Review

**DOI:** 10.7150/ijms.64984

**Published:** 2022-01-01

**Authors:** Fitri Fareez Ramli, Adli Ali, Nurul 'Izzah Ibrahim

**Affiliations:** 1 Department of Pharmacology, Faculty of Medicine, Universiti Kebangsaan Malaysia, 56000 Cheras, Kuala Lumpur, Malaysia.; 2 Cardiovascular Health Research Group, Faculty of Medicine, Universiti Kebangsaan Malaysia, 56000 Cheras, Kuala Lumpur, Malaysia.; 3 Department of Pediatrics, Faculty of Medicine, Universiti Kebangsaan Malaysia, 56000 Cheras, Kuala Lumpur, Malaysia.; 4 Infection and Immunology Health and Advanced Medicine Cluster, Universiti Kebangsaan Malaysia, 56000 Cheras, Kuala Lumpur, Malaysia.

**Keywords:** molecular-signaling pathway, ginsenoside Rb1, ginsenoside Rb2, ginsenoside Rb3, myocardial ischemia-reperfusion injury

## Abstract

Reperfusion injury following myocardial ischemia remained a challenge for optimal treatment of myocardial infarction. Ginsenosides Rb (G-Rb), the primary components of ginsenoside, have been reported to exert cardioprotective effects via numerous mechanisms. G-Rb1 mediate cardioprotective effects via various signaling pathways, including mitochondrial apoptotic pathway, PI3K/Akt/mTOR, HIF-1α and GRF91, RhoA, p38α MAPK, and eNOS. G-Rb2 activates the SIRT-1 pathway, while G-Rb3 promotes both JNK-mediated NF-κB and PERK/Nrf2/HMOX1. Generally, ginsenosides Rb1, 2, and 3 modulates oxidative stress, inflammation, and apoptosis, contributing to the improvement of structural, functional and biochemical parameters. In conclusion, G-Rb, particularly G-Rb1, have vast potential as a supplement in attenuating reperfusion injury. Translation into a clinical trial is warranted to confirm the beneficial effects of G-Rb.

## Introduction

Myocardial infarction (MI) is one of the manifestations of ischemic heart disease (IHD). According to the Global Epidemiology study, more than a hundred million people were estimated to suffer from IHD worldwide 1. MI causes irreversible damage to the heart muscle due to the oxygen-deficient condition, contributing to impaired diastolic and systolic functions 2. Immediate restoration of cardiac perfusion is pivotal to salvage viable tissues from the ongoing insults. Currently, various reperfusion techniques, including coronary artery bypass grafting, thrombolytic therapy, and percutaneous coronary intervention, are available and have been shown to considerably improve the survival rate of patients with MI 3. However, reperfusion following MI often exacerbates myocardial damage, worsening cardiac dysfunction and structural impairment attributable to numerous cardiac pathologies 4, 5.

A burst of reactive oxygen species (ROS) from the mitochondrial respiratory chain may initiate various pathological events during the ischemia-reperfusion period. During ischemia, the mitochondrial calcium concentration becomes prominent, and this event is attributable to calcium influx via mitochondrial calcium uniporter, ryanodine receptor type 1 channels and low conductance mitochondrial permeability transition pore (mPTP) 6. Together with the pooling of calcium ions in mitochondria, ROS accumulation causes sustained opening of mPTP, particularly during reperfusion, promoting mitochondrial dysfunction and impaired cellular homeostasis 6. Other than that, myocardial ischemia-reperfusion injury (MIRI) promotes both regulated and non-regulated cell death, such as apoptosis, ferroptosis, pyroptosis, necrosis and autophagy 7. MIRI which can be described as distinct structural and functional impairments of the heart following the restoration are associated with significant changes such as oxidative stress, inflammation and apoptosis and therefore, additional therapy is pivotal to optimize reperfusion treatment 4, 5.

Over the past decade, many studies have been conducted on the individual ginsenoside, focusing on the molecular-signaling pathways in MIRI 8-10. There are more than dozens of ginsenosides, but the main type of ginsenosides (G) is Rb, including Rb1, Rb2, and Rg1 that account for more than 80% of total ginsenosides 11. Cumulative evidence indicates the beneficial cardioprotective effects of G-Rb when administered prior to experimental induction of MIRI 8-10, 12-24. The term preconditioning refers to endogenous mechanism stimulated by G-Rb given before MIRI via activations of kinases and other mediators that confer more resistant heart condition toward MIRI. Contradictorily, the mechanism of ischemic postconditioning-induced protection occurs following MIRI, involving the activation of reperfusion injury salvage kinase (RISK) pathway and subsequently causes the inhibition of mPTP 25. However, the application of G-Rb for postconditioning in animal model is scanty, which might be due to complications following the left coronary artery ligation 26. To the best of our knowledge, there were no clinical studies involving the ginsenoside Rb in MIRI have been conducted. Only a few clinical trials that have been done using G-Rb including pharmacokinetics studies, insulin secretion and plasma glucose levels as well as motility of human sperm in semen samples. Lack of clinical studies that involve ginsenosides are associated with their low bioavailability (poor water solubility and biomembrane permeability, instability in gastrointestinal tract, and high metabolic rate in the body 27. Previous reviews on the effects of ginsenosides in MIRI were broad, discussing various types and aspects of ginsenoside in general 11, 28. Therefore, in the present review, we aimed to focus on the mechanism of major components of ginsenosides (Rb), emphasizing the molecular-signaling pathway responsible for the cardioprotective effects against MIRI specifically in *ex vivo*,* in vivo*, and* in vitro* models.

## Ginsenoside

**Figure 1:**
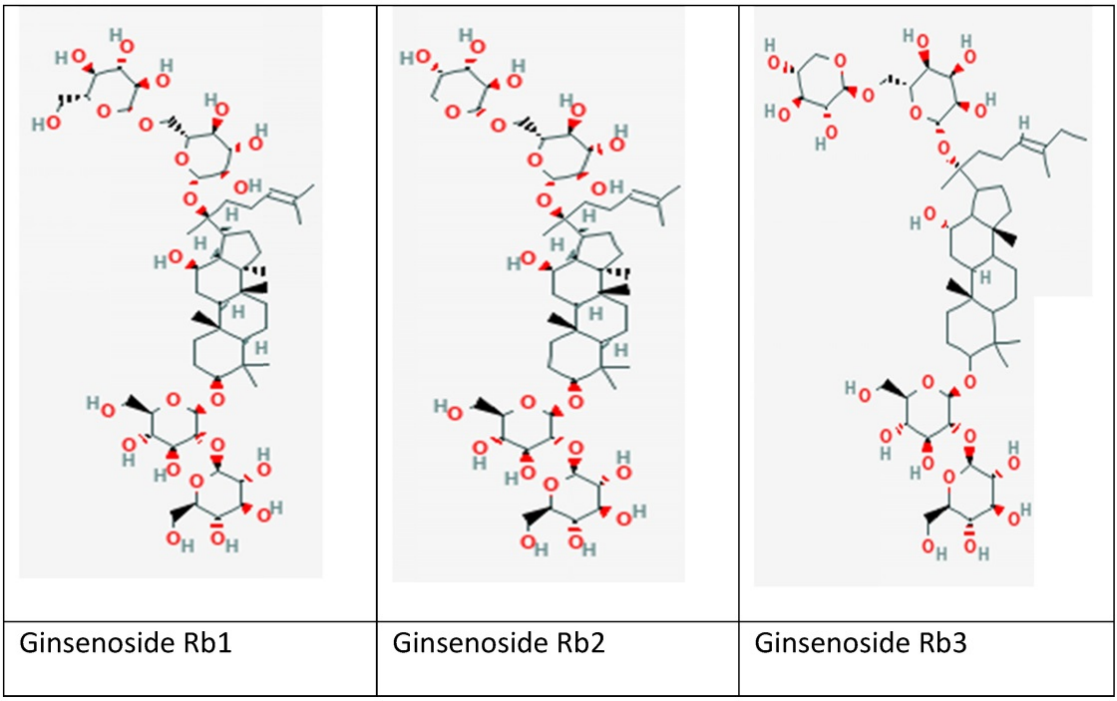
Chemical structure of selected ginsenosides included in this review; (a) Rb1; (b) Rb2 and (c) Rb3. Source: https://pubchem.ncbi.nlm.nih.gov/

Ginseng, a perennial plant belonging to the Araliaceae family (Panax genus), has been extensively studied for its antioxidant defense mechanism with potential protection against MIRI 37-39. Ginseng is particularly used in Asian countries for thousands of years to maintain body homeostasis and energy enhancement 37, 40, 41. It is a valuable folk medicine in East Asian countries such as Korea, China and Japan. Among approximately 20 different species of ginseng in the genus, *P. ginseng* (Korean ginseng), *Panax notoginseng* (Chinese ginseng), *Panax japonicum* (Japan ginseng), and *Panax quinquefolius* (American ginseng) are the most commonly used as medicines 42. Various natural products that can be used routinely in the diet have demonstrated the positive effects against MIRI by aiding to control and prevent clotting 43, 44. The natural products such as ginseng are being preferred by people as the side effects are generally fewer compared to existing synthetic drugs disease 43. Apart from antioxidant properties, ginseng has been reported of possessing other biological activities, including anti-inflammatory, anti-ageing, anti-diabetic, anti-cancer, wound and ulcer healing activity, as well as to treat cardiovascular diseases 45. Ginseng mainly comprised of various bioactive monomers, such as saponins, fatty acids, polysaccharides, and mineral oils. Among all the monomers in ginseng, its pharmacological activities are attributed mainly to saponins, also known as ginsenosides 46. The ginsenosides are extracted from roots, fruits, stems and leaves of ginseng. They are classified into three types accordingly to chemical compositions and configurations: (i) Panaxadiol group (e.g., Rb1, Rb2, Rb3, Rc, Rd, Rg3, and Rh2), (ii) Panaxtriol group (e.g., Re, Rg1, Rg2, and Rh1), and (iii) Oleanolic acid group (e.g., Ro) 47. The ginsenosides were named alphabetically based on migration distance at the thin layer chromatography (TLC) such as Rb1, Rb2, Rb3, Rc and Re 48. As ginsenosides are associated with anti-oxidation, anti-inflammation and cardioprotective effects, they are commonly used in individuals with cardiovascular risk factors such as cigarette smoking, hypercholesterolemia, hypertension, and diabetes 42. Current studies of G-Rb (Figure 1) reported remarkable cardioprotective effects in MIRI conditions. Different components have been shown to affect the heart via numerous molecular-signaling pathways.

### Ginsenoside Rb1

**Figure 2:**
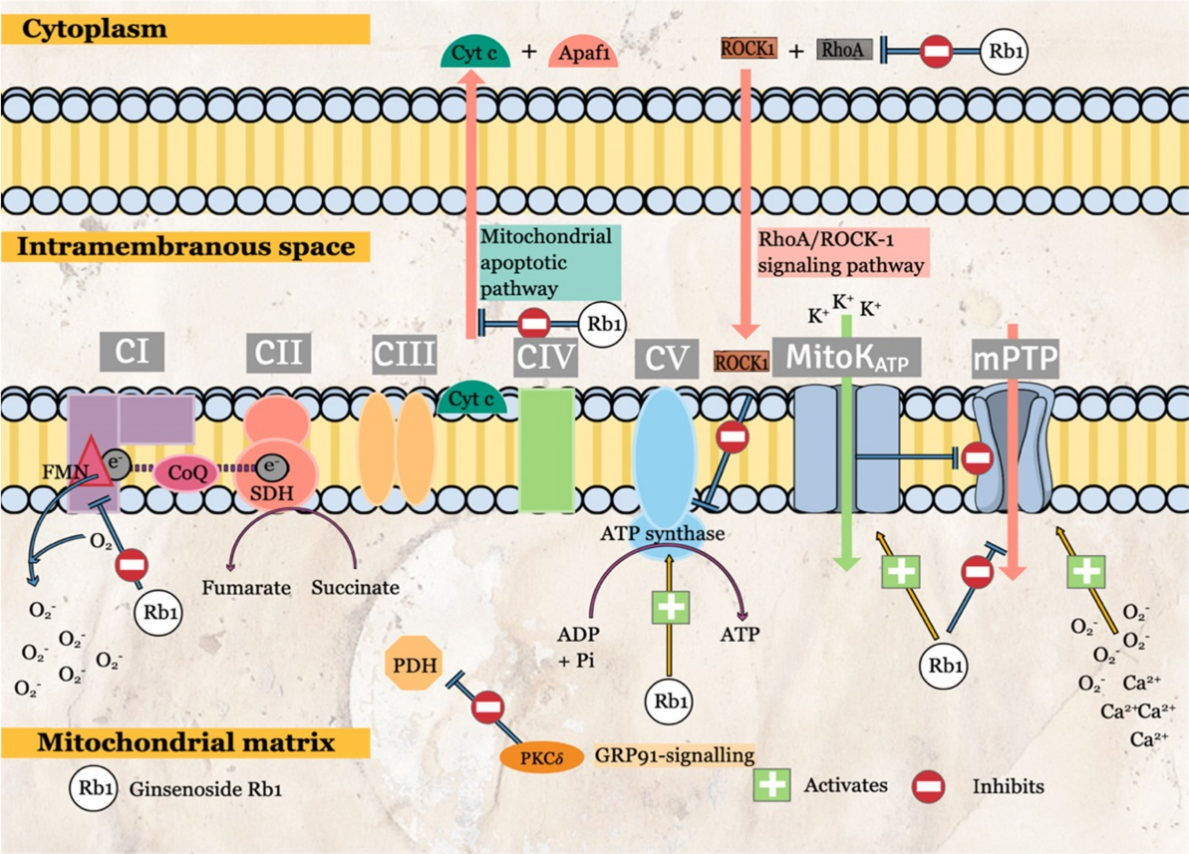
The molecular-signaling pathways of ginsenoside in reperfusion state during ischemia-reperfusion (I/R) injury in mitochondria.

G-Rb1 is the most widely studied components among G-Rb monomers. Numerous cardioprotective effects have been reported that is mediated by various molecular-signaling pathways. Among the prominent effects of G-Rb1 are related to protective mitochondrial effects. G-Rb1 attenuates mitochondrial ROS production via the specific inhibition of mitochondrial complex I (Figure 2) 9. The transient G-Rb1 blockade allows mitochondrial complex I to remain in a deactivated form, inhibiting NADH dehydrogenase activity during reperfusion. The inhibition is specific to mitochondrial complex I as G-Rb1 has no significant effects on mitochondrial complex II and IV activities 9. As a result, mitochondrial ROS production is significantly reduced. The effects of G-Rb1 on mitochondrial complex I is further supported by proteomic analyses that demonstrated the significant effects on proteins that regulate NADH dehydrogenase and oxidoreductase activities 9.

**Figure 3:**
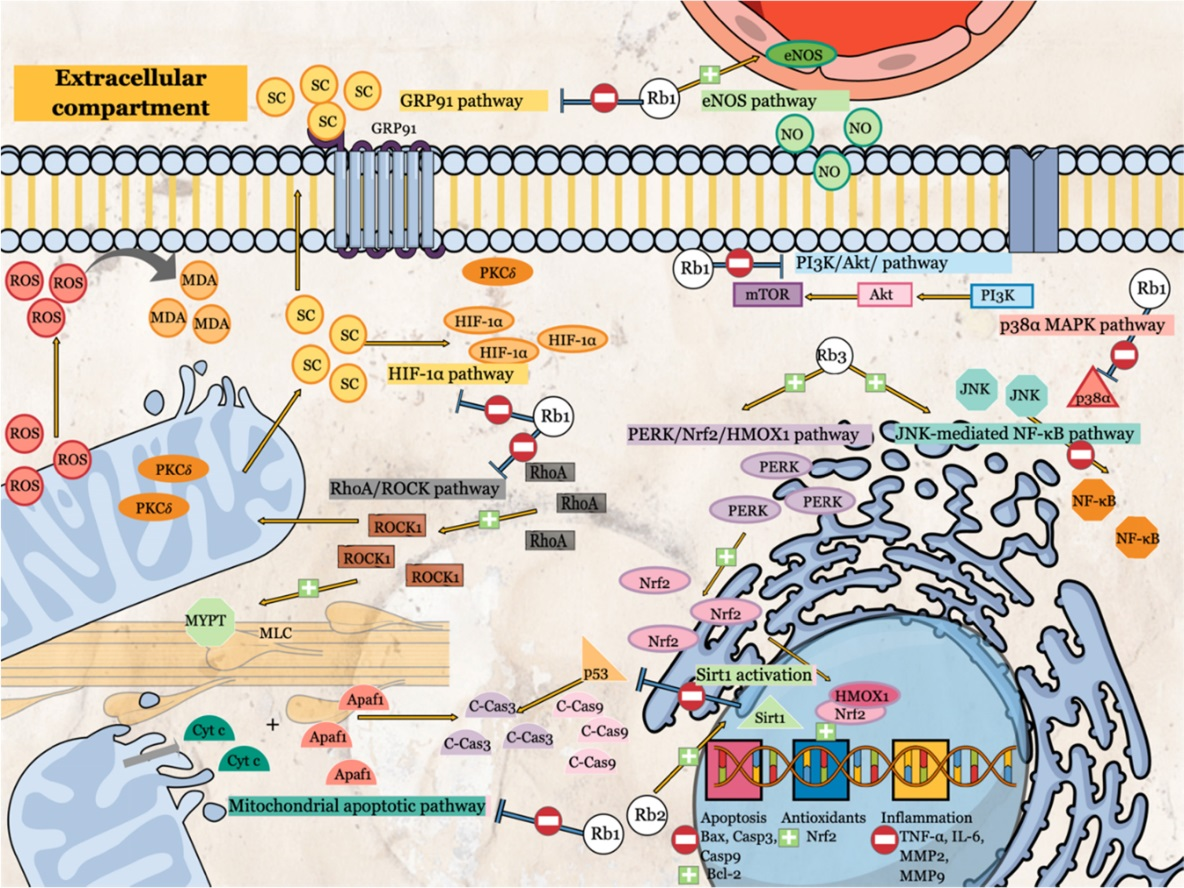
Molecular signaling pathways of ginsenosides that protect cardiomyocytes against myocardial ischemia/reperfusion injury (MIRI).

Other than that, G-Rb1 regulates energy metabolism via the interaction with RhoA. MIRI causes ATP depletion, probably due to RhoA/ROCK (Rho-associated coiled-coil containing protein kinase-1) activation, which reduces the expressions of ATP5D subunit (proteins and mRNA) and ATP synthase activity. Cui, Pan 12 had reported the significant G-Rb1 reversal effects in ATP production, ATP5D expressions and ATP synthase activity via inhibition of RhoA and its downstream effectors. Although no specific inhibitor was used in this study, the surface plasmon resonance has demonstrated the binding of G-Rb1 to RhoA. Other than energy regulation, ROCK-1 interacts with RhoA to cause the phosphorylation of both myosin light chain (MLC) and MLC phosphatase (MYPT-1) 12, stimulating smooth muscle contraction. Pre-conditioning with G-Rb1 inhibits the activation of MLC and MYPT-1 (Figure 3). Another target of G-Rb1 is the MitoKATP channel. The channel is embedded in the inner membrane of mitochondria and play an essential role in the regulation of MMP and mitochondrial volume. G-Rb1 partly protects mitochondrial damage via activation of this channel 34.

Li, Qian 15 reported that G-Rb1 administration before experimental MIRI attenuated the phosphorylation of p38α, thus inhibiting the production of pro-inflammatory and pro-apoptotic mediators (Table 1). Moreover, co-administration of p38α MAPK activator with G-Rb1 reversed the protective effects of G-Rb1 15. p38α is the primary isoform presence in human and rodent myocardium, and its activation is associated with contractile dysfunction and cardiomyocyte death 15.

Also, PI3K/Akt signaling pathway and its downstream pathway of mTOR regulate cellular apoptosis. G-Rb1 has been reported to modulate these pathways via phosphorylation of Akt and mTOR, resulting in reduced pro-apoptotic markers, apoptosis, infarct size, and cardiac enzymes leakage 14, 20, 21. Concurrently, a significant increase in anti-apoptotic markers is observed. In addition, G-Rb1 also shows similar cardioprotective effects in experimental MIRI in the presence of endocrine abnormalities, such as diabetes mellitus 21. Co-administration with wortmannin (non-specific PI3K inhibitor) or rapamycin (mTOR inhibitor) confirm the involvement of the PI3K/Akt/mTOR signaling pathway in G-Rb1 protective effects as evidenced by the reversal of G-Rb1 effects 14, 20, 21. Furthermore, PI3K/Akt pathways have been proposed to mediate other downstream effects, such as protein kinase C activation and nitric oxide production 20.

Next, G-Rb1 provides cardioprotection partly through endothelial nitric oxide synthase (eNOS) activation that increases nitric oxide (NO) bioavailability 22. NO is a potent endogenous vasodilator and provides various protective factors at physiologic levels, including the inhibition of superoxide production, enhancement of endogenous antioxidative capabilities, attenuating pro-inflammatory cytokines activation, inhibition of inflammatory cells activation, downregulation of vascular smooth muscle cells proliferation and activation, as well as suppression of production and secretion of extracellular matrix metalloproteinases while increasing the production of tissue inhibitor of matrix metalloproteinases 49. In a diabetic model, Xia, Zhao 22 had reported the significant role of G-Rb1 on NO production and eNOS expression in regulations of oxidative stress as confirmed by the reversal to pathological conditions (cardiac enzymes leakage, production of lipid peroxidation product, reduction in antioxidant and NO levels, infarct size) when L-NAME, an inhibitor for eNOS was combined with G-Rb1.

Furthermore, G-Rb1 promotes cardioprotection via the regulation of hypoxia inducible factor-1α (HIF-1α) and G-protein-coupled receptor GPR91 signaling pathway 50. Li, Yang 50 had conducted a study in three different models (*in vivo, ex vivo* and* in vitro*) exposed to either high-fat diets in the first model or palmitate for the latter models. Persistent exposure to high-fat diets in *in vivo* model or stimulation by palmitate (saturated fatty acid) in *ex vivo* and *in vitro* models cause succinate accumulation secondary to elevated β-oxidation of fatty acid in mitochondria 50. Subsequently, succinate inhibits pyruvate dehydrogenase (PDH) activity via two signaling pathways. Firstly, intracellular succinate enhances the expression of HIF-1α, a transcriptional factor of pyruvate dehydrogenase kinase 4 (PDK4), essential in energy metabolism in anoxic conditions. HIF-1α can induce pro-apoptotic and inflammatory mediators in ischemic reperfusion injury 50. PDK4 phosphorylates PDH, resulting in PDH inactivation. Secondly, intracellular succinate may diffuse into the extracellular compartment and interact with GPR91 on the cell membrane to initiate downstream pathways involving protein kinase δ (PKCδ). Translocation of PKCδ into mitochondria deactivates PDH activity, leading to disruption of glucose oxidation. Dual impacts of succinate via two signaling pathways contribute to marked attenuation of PDH activities. Elevated fatty acid oxidation consumes a significant amount of oxygen 50. Moreover, inhibition of PDH interrupts glucose oxidation. These events lead to reduced efficiency of cardiac energy production that increases the susceptibility of cardiomyocytes to MIRI. G-Rb1 has been reported to regulate PDH activities via inhibition of β-oxidation, resulting in reduced succinate accumulation and subsequent events 50. The importance of PDH activities in energy metabolism, particularly in MIRI, is supported by Ussher, Wang 51 findings that showed a significant reduction in infarct size when PDK inhibitor was given or PDK4 deficient mice compared to wild type. However, the lack of specific inhibitors related to HIF-1α- and GRP91- signaling pathways used in the study by Li, Yang 50 may not be able to explain the effects of G-Rb1 on these pathways fully.

### Ginsenoside Rb2

G-Rb2 activates Sirtuin-1 (SIRT1) to promote cardioprotective effects in MIRI conditions 35. Xue, Fu 35 had confirmed that the G-Rb2 mechanism is attributable to SIRT1 activation as the addition of SIRT1 inhibitor, EX527, in a group of MIRI+Rb2 had significantly reversed the cardioprotective parameters related to oxidative stress (gp91phox, malondialdehyde, superoxide), antioxidant enzymes levels (catalase (CAT), superoxide dismutase (SOD), glutathione peroxidase (GSH-Px), inflammatory cytokines (IL-1β, IL-6, TNF- α), as well as pro- (Bax protein, cleaved caspase -3 and -9) and anti-apoptotic markers (Bcl-2). SIRT1 is a class III histone deacetylase essential for various cellular processes, such as apoptosis and autophagy 52. Enhancement of SIRT1 results in the inhibition of p53 acetylation, reducing the downstream apoptotic pathway 35. Consequently, the reduction in oxidative stress and inflammation by SIRT1 also lead to significant structural (infarct size), functional (LVEF, LVFS), and biochemical (AST, CK-MB, LDH) improvement of the heart affected by MIRI 35.

Similarly, Liu, Jiang 17 also reported similar improvements when G-Rb2 was given in combination with G-Rb3 in terms of antioxidant enzymes (CAT, GSH peroxidase, SOD), Bcl-2, and functional parameters (SBP, DBP, HR, LVSP, +dp/dt max). In contrast, remarkable reductions were observed in infarct size, apoptosis (index and pro-apoptotic markers), inflammatory markers (IL-6, TNF-α) and cellular enzymes leakage (AST, CK-MB, LDH). On the other hand, There were no significant differences between a combined preparation of G-Rb2/3, G-Rb3 alone and diltiazem (served as a positive control) 17. The results indicated similar mechanisms and potentially shared cardioprotective molecular-signaling pathways.

Although the evidence on how G-Rb2-induced SIRT1 activation mediates cardioprotection is still limited, several mechanisms can be proposed. SIRT1 activation reduces the expression of mitochondrial uncoupling protein (UCP) 2 via direct interaction with its promoter. Deng, Wang 53 reported that SIRT1 activation via either overexpression of SIRT1 or knockdown of UCP-2 in cardiomyocytes resulted in increased in cell viability, potentially secondary to enhanced ATP synthesis and improvement of cellular acid-base balance. Another potential mechanism is the endothelial nitric oxide synthase (eNOS) upregulation by SIRT1. Ding, Lei 54 had demonstrated a remarkable enhancement of eNOS activity in diabetic rats transfected with SIRT1, which was attenuated when NOS inhibitor was added.

Other than that, stimulation of the peroxisome proliferator-activated receptors gamma co-activator-1 alpha (PGC-1α) activities by SIRT1 is another potential mechanism related to cardioprotective effects via the SIRT1 pathway 55. In a study conducted by Wang, Cao 55, SIRT1 deletion significantly attenuate the expression of PGC-1α, an essential transcription factor for many genes, including nuclear factor erythroid 2-related factor 2 (Nrf2). Subsequently, the expression of antioxidants enzymes SOD and heme-oxygenase 1 (HO-1) reduced, contributing to oxidative stress and eventually cell death 55.

### Ginsenoside Rb3

Two molecular-signaling pathways related to cardioprotective effects of G-Rb3 are JNK-mediated NF-κB and PERK/Nrf2/HMOX1. G-Rb3 inhibits the JNK-mediated NF-κB activity 8. NF-κB presence in combination with IκB-α in the cytoplasm 8. The phosphorylation of IκB-α activates NF-κB, promoting its translocation into the nucleus and binding to specific regions to activate inflammatory response 8. G-Rb3 activates JNK via phosphorylation which attenuates the activation, translocation, and DNA binding of NF-κB components, reducing oxidative stress and inflammatory markers such as superoxide, IL-6, TNF-α, MCP-1, MMP-2 and MMP-9 8. The use of phospho-JNK inhibitor (SP600125) shows similar changes to G-Rb3 in terms of cell viability, apoptosis, and NF-κB activity 8. Although similar, no direct activator was utilized in the study conducted. In contrast to JNK, Ma, Liu 8 reported that the other upstream pathways of NF-κB such as Akt/Foxo3a, p38, and ERK are not mediated by G-Rb3.

Other than that, G-Rb3 increases the phosphorylation of PERK, an essential factor in ATF4-mediated endoplasmic reticulum stress, resulting in the activation of Nrf2 following MIRI 19. Nrf2 present in the cytoplasm as an inactive form that associates with keap1 and Cullin3 proteins 56. Once Nrf2 activated, this factor is translocated into the nucleus to interact with other factors, such as HMOX1, which leads to the enhancement of antioxidant-related genes transcription 56. CAT, GSH-Px, HO-1, SOD, and NADH: quinone oxidoreductase I are the proteins that are regulated by Nrf2 upon its interaction with the antioxidant response element 56. Increasing transcriptional activity of antioxidants-related genes boosts intracellular antioxidants levels (total equivalent antioxidants capacity), attenuating oxidative stress (ROS) 19. Subsequently, G-Rb3 causes remarkable reductions in apoptosis and infarct size. Also, Sun, Yu 19 confirmed the involvement of this pathway by using GSK2656157 (PERK inhibitor) and ML385 (Nrf2 inhibitor), which resulted in the reversal of G-Rb3 mediated effects.

## Safety and Toxicity of Ginsenoside Rb

In a previous study by Xiong et al. (2010), ginsenoside Rb1 given intraperitoneally to high-fat diet (HFD)-induced obese rats for four weeks has shown no obvious health problems (no side effects such as diarrhea) 57. This may have indicated that ginsenoside Rb1 is safe to be consumed in a chronic period without causing side effects. Apart from that, an oral administration of Rb1 and Rb2 at 50, 100, and 200 mg/kg for 7 days to carbon tetrachloride (CCL4)-induced hepatotoxicity in rats showed no toxic effects, as indicated by the insignificant alteration to the organ weights, hematological and serum clinical parameters. Additionally, partial protection was observed for the elevated serum glutamate pyruvate transaminase (SGPT) and serum glutamic-oxaloacetic transaminase (SGOT) following the CCL4 induction. Meanwhile, histologically, an improvement in liver vacuolization and lymphoid cell aggregation were also observed following the oral supplementation of Rb1 and Rb2 58. These results may have indicated that the Rb 1 and Rb 2 may partially reverse the CCL4-hepatotoxicity induced in animal model.

## Conclusion and Perspectives

All three ginsenosides (Rb1, Rb2, Rb3) share similar properties in cardioprotection via the mediation of oxidative stress, inflammation, and apoptosis, as indicated in the preclinical studies. Pre-treatment with G-Rb improves numerous structural, biochemical, and functional parameters reported in *ex vivo*,* in vivo*, and* in vitro* models via numerous molecular-signaling pathways, including JNK-mediated NF-κB, PERK/Nrf2/HMOX1, SIRT1 activation, eNOS, PI3K/Akt/mTOR, p38α MAPK, HIF-1α and GPR91, RhoA, and mitochondrial apoptotic pathways. However, further studies should be conducted to address the limitation of the current evidence. Most of the included studies were primarily focused on G-Rb1. More studies are required to elucidate the molecular-signaling pathways for G-Rb2 and G-Rb-3. Determination of other molecular-signaling pathways allows us to understand whether all G-Rb share similar or distinct mechanisms with synergistic effects. Secondly, there was only one study comparing G-Rb3 and a combination of GRb2/3 to date. Comparison between specific G-Rb or a combination of those is necessary to ascertain which G-Rb1 is the most effective or whether the combination of G-Rb components may provide additional benefits for cardioprotection. Moreover, the use of specific inhibitors may be limited in some studies. Although certain studies had utilized positive control to compare the effects with G-Rb, the molecular-signaling pathways contributing to cardioprotective effects may not be similar, albeit similar effects on several parameters. Other than that, determining the most optimal timing for G-Rb administration to provide maximum cardioprotection may be beneficial for future clinical studies. Despite several limitations, G-Rb has enormous potential to become a supplement for reducing the impact of reperfusion injury following MI in the future.

## Figures and Tables

**Table 1:** The effects of specific ginsenoside Rb on oxidative stress, antioxidants, inflammation and apoptosis parameters as well as the molecular-signaling pathways.

Study	Study type	Oxidative stress		Antioxidants				Inflammation			Apoptosis						Molecular-signaling pathway (+/-)
		ROS	MDA	SOD	CAT	GSH-Px	TEAC	TNF-α	IL-1 β	IL-6	Casp3	Casp9	Bax	Bcl2	Bax/ Bcl2	AI	
Ginsenoside Rb1																	
Jiang et al. 9	*In vivo*	↓														↓	Mitochondrial apoptotic pathway (-)
	*In vitro*	↓											↓	↑			
Li et al 2020 14	*In vivo*										↓ ⇡^R^		↓ ⇡^R^	↑ ⇣^R^			Mechanistic target of rapamycin (mTOR) (+)
Zhang et al 2019 34	*In vitro*	↓ ⇡^5-HD^									↓ ⇡^5-HD^					↓ ⇡^5-HD^	MitoK_ATP_ channel regulation (+)
Cui et al. 2017 12	*In vivo*										↓	↓			↓	↓	RhoA/ROCK (-)
Li et al 2016 15	*In vivo*							↓ ⇡^Ani^			↓ ⇡^Ani^						p38α MAPK (-)
Xia et al 2011 22	*In vivo*		↓ ⇡^L-NAME^	↑ ⇣^L-NAME^													eNOS (+)
Wu et al. 2011 21	*In vivo*										↓^a^ ⇡^Wor^					↓ ⇡^Wor^	PI3K/Akt (+)
Guan et ak. 2002 13	*In vivo*															↓	
Ginsenoside Rb2																	
Xue et al. 35	*In vivo*	↓ ⇡ ^EX527^	↓ ⇡ ^EX527^	↑ ⇣^EX527^	↑ ⇣^EX527^	↑ ⇣^EX527^		↓ ⇡ ^EX527^	↓ ⇡ ^EX527^	↓ ⇡ ^EX527^	↓ ⇡ ^EX527^	↓ ⇡ ^EX527^	↓ ⇡ ^EX527^	↑ ⇣^EX527^	↓	↓ ⇡ ^EX527^	Sirtuin 1 (SIRT1) (+)
Ginsenoside Rb3																	
Sun et al. 2019 19	*In vivo*						↑ ⇣^ ML385^									↓ ⇡^ ML385^	PERK/Nrf2/ HMOX1 (+)
	*In vitro*	↓ ⇡^ GSK^ ⇡^ ML385^					↑						↓ ⇡^ GSK^ ⇡^ ML385^	↑ ⇣^GSK^ ⇣^ ML385^		↓ ⇡^ GSK^ ⇡^ ML385^	
Ma et al 2014 8	*In vitro*	↓						↓								↓ ⇡^ LPS^	JNK-mediated NF-κB (+)
Liu et al. 2014 10	*In vivo*		↓	↑				↓		↓			↓	↑		↓	
Shi et al 2011 18	*In vivo*		↓	↑													
Ginsenoside Rb2/3																	
Liu et al. 2020 17	*In vivo*		↓	↑	↑	↑		↓		↓	↓		↓	↑	↓	↓	

↑ or ↓: increased or decreased in MIRI+G-Rb compared to MIRI, respectively. (+) activates; (-) inhibits. 5-HD: a specific blocker of MitoK_ATP_; Anisomycin: p38α MAPK agonist; GSK2656157: PERK inhibitor; LPS: NF-κB activatior; ML385: Nrf2 inhibitor; Rapamycin: mTOR inhibitor; SP600125: phospho-JNK inhibitor. *Abbreviations:* Bax: Bcl-2 Associated X protein; Bcl-2: B-cell lymphoma-2; Casp: caspase; CAT: catalase; GSH-Px: glutathione peroxidase; MDA: malondialdehyde; nrf2: nuclear factor erythroid 2-related factor 2; ROS: reactive oxygen species; SOD: superoxide dismutase
